# Prevalence of dirofilariasis in shepherd and stray dogs in Iranshahr, southeast of Iran

**DOI:** 10.1007/s12639-019-01096-5

**Published:** 2019-02-14

**Authors:** Davood Anvari, Dariush Saadati, Abolghasem Siyadatpanah, Shirzad Gholami

**Affiliations:** 1 Student Research Committee, Department of Parasitology, Mazandaran University of Medical Sciences, Sari, Iran; 2 Department of Nutrition and Animal Breeding, Faculty of Veterinary Medicine, University of Zabol, Zabol, Iran; 3 Department of Parasitology, Toxoplasmosis Research Center, Mazandaran University of Medical Sciences, Sari, Iran

**Keywords:** *Dirofilaria immitis*, Dirofilariasis, Dog, Southeast of Iran, Iranshahr

## Abstract

Dirofilariasis is a zoonotic parasitic disease, which its cause *Dirofilaria immitis*, a nematode transmitted by insects and a worldwide dissemination. Dogs and cats are the main hosts of this parasite. Therefore, this study was conducted to determine the prevalence of *D. immitis* in shepherd and stray dogs in Iranshahr city, southeast of Iran. In this study 49 shepherd dogs and 50 stray dogs selected randomly and the blood samples were taken and sent to parasitology laboratory. Thin and thick thin blood samples were prepared and stained with Giemsa method and modified knott method. The obtained data were analyzed by SPSS 18 statistical software. The overall prevalence of parasite was 30.3%, seven of 49 shepherd dogs (14/3%) and twenty three of 50 stray dogs (46%) were positive. From 30 positive samples, 14.3% of shepherd dogs and 50% of stray dogs were male, and 44.7% of stray dogs were female. 14% of infected shepherd dogs and 38.5% of infected stray dogs were indigenous, and 16.7% of infected shepherd dogs and 72.7% of infected stray dogs were hybrids. There was no statistically significant relationship between the prevalence of *D. immitis* with age, gender, breed and usage of anti-parasitic drugs. Due to the relatively high contamination of dogs in the city of Iranshahr with this parasite, veterinarians and public health professionals should be considered necessary and preventive measures in relation to this disease.

## Introduction

Dirofilariasis is a common disease between humans and animals, which causes by *Dirofilaria immitis* (heartworm), a nematode that can be transmitted by insects and it spread worldwide. Many domestic animals, especially dogs and cats, are the main host of this parasite (Atkins 2003). Human may be infected with the larval stage as an intermediate host, and so far several human cases have been reported from the United States, Japan and Iran (Ettinger and Feldman 2005). Adult nematode forms are seen in the right pulmonary and right ventricular arteries of dogs and the larval stage of the parasite (microfilariae) can be transmitted to the vertebrate hosts such as human and animals through the mosquito bite (*Anopheles* and *Culex*). Dirofilariasis in dogs can cause clinical symptoms such as shortness of breath, continuous cough, intolerance to severe physical activity, congestive heart failure, hemoptysis, intravascular hemolysis, pulmonary thromboembolism, ascites, loss of appetite and weight. Dirofilariasis disease is one of the most important zoonosis between humans and carnivores in tropical and subtropical regions (Vieira et al. 2014) and in dogs if not treated, it can be fatal (Taylor et al. 2007). Although human is an accidental host for *D. immitis* nematode, pulmonary dirofilariasis and ectopic infections have been reported frequently from humans. So far, more than 1700 human cases of dirofilariasis have been recorded worldwide, with more than 370 cases of pulmonary disease occurring among them. So wherever dog dirofilariasis exists, humans are also at risk of contamination. Most human infections are asymptomatic. This parasite produces coin-like lesions in the lungs, which have little pathological significance (Ciferri 1982; Echeverri et al. 1999; Montoya-Alonso et al. 2010; Robinson et al. 1977; Shaw and Day 2005; Simon et al. 2012; Taylor et al. 2007). The lesions created by this parasite, in spite of benign condition, have great importance to humans because spherical granulomas in the underlying curvature may be mistaken for radiography with primary or metastatic tumors (pulmonary neoplasia) leading to unnecessary procedures for diagnosis (tracheotomy and biopsy) and treatment (Brown and Barker 2007; Etinger 2000). Also, cardiovascular injuries, intraocular and posterior ocular infections, peritoneal cavity infections, chest pain, infection of male reproductive organs, and rare cases of meningoencephalitis have been reported from humans (Kronefeld et al. 2014). This disease has a worldwide spread. In Iran, for the first time, *D. immitis* is reported in a dog in 1969 (Eslami 2008). Subsequently, it has been reported in other different regions of Iran such as Tehran, Tonekabon, Kermanshah, Ardebil, Mazandaran, Semnan, Kerman, Fars, Golestan, and Tabriz (Akhtardanesh et al. 2011; Bohloli Oskoii et al. 2013; Bokai et al. 1998; Hosseinzadeh Varjoy et al. 2016; Jafari et al. 1996; Khedri et al. 2014; Malmasi et al. 2011; Meshki and Eslami 2001; Ranjbar-Bahadori et al. 2011; RanjbarBahadori and Hekmatkhah 2007; RanjbarBahadori et al. 2005; Sadighian 1969; Sadjjadi et al. 2004).

In southeast of Iran, only in Zabol city in Sistan region has reported a seroprevalence of this parasite of 27.5% in stray and domesticated dogs by Khedri et al. (2014). Therefore, the present study was conducted to determine the prevalence of *D. immitis* in shepherd and stray dogs in Iranshahr (south of Sistan and Balouchestan province), southeast of Iran.

## Materials and methods

In this study, in order to determine the prevalence of dirofilariasis, 49 shepherd dogs from Iranshahr villages in Balouchestan and 50 stray dogs randomly collected by the cooperation of municipality during the summer of 2017, and their blood samples were collected. In terms of age, the stray dogs were divided into two groups including under 1 year and upper 1 year old, moreover shepherd dogs were divided into 3 groups including under 1 year, 1–3 years and upper 3 years old. Blood sampling from dogs after taken permission from owners of shepherd dogs and clinical examinations was performed and their profiles including sampling date, age, gender, breed and use or non-use of anti-parasitic drugs was recorded. At this stage, 1 cc of blood was taken from the cephalic vein or saphenous dogs and immediately transferred to a tube containing 9 cc formalin 2% and then shaken slowly to allow hemolysis of the red blood cells. The specimens were sent to the laboratory for Modified Knott test and the samples were examined for the presence of *Dirofilaria immitis* microfilariae. In this way, the mixture of 1 cc of blood and 9 cc of formalin 2% was centrifuged at 1500 rpm for 5 min. Then the top solution of the tube was slowly discharged and added one to two drop of Methylene blue to the precipitate and transferred with a pipette to the slide and the slides were observed using an optical microscope with 40 × and 10 × lens for the presence of microfilariae of *Dirofilaria immitus*. Also, the samples were stained with Giemsa method on thick blood slides. *Dirofilaria immitis* is subjected to other microfilariae of dogs (*Dipetalonema reconditum* and *Dirofilaria repens*) with the following characteristics: The anterior end of the microfilariae of *D. immitis* is gradually narrowed, its posterior end is straight and sheathed and moves slowly (Eslami 2008).

The results were entered into the questionnaire and each dog’s information (e.g. age, gender, breed, and usage of anti-parasitic drug) was entered into spss18 software where the data were analyzed by Chi square statistical and Fisher’s exact tests. The confidence level of 95% and *P* value < 0.05 were considered as significant level.

## Results

In this study, out of 99 dogs, the overall prevalence of *Dirofilaria immitis* was 30.3% (30/99). 14.3% (7 cases) of shepherd and 46.0% (23 cases) of stray dogs was infected. The difference between the prevalence of *D. immitis* in shepherd and stray dogs was statistically significant (*P* < 0.001) (Table 1).

**Table 1:** The factors related to the prevalence of dirofilariasis in shepherd and stray dogs

Breed of dog	Shepherd dogs			*P* value	Stray dogs			*P* value
Subgroups	No. sample	Positive	%		No. sample	Positive	%	
*Age*								
Under 1 year	5	0	0.0	0.006	10	4	40.0	0.67
1–3 year	36	3	8.3	–	–	–	–	
Upper 1 year	–	–	–		40	19	47.5	–
Upper 3 year	8	4	50.0	–	–	–	–	
*Gender*								–
Male	49	7	14.3	–	12	6	50.0	0.75
Female	–	–	–		38	17	44.7	–
*Breed*								
Native	43	6	14.0	1	39	15	38.5	–
Hybrid	6	1	16.7		11	8	72.7	0.044
*Usage of anti-parasitic drug*								
Yes	28	4	14.3	1	–	–	–	–
No	21	3	14.3		50	23	46.0	1

The results of this study showed that the prevalence of *Dirofilaria immitis* increases with age. Chi square test showed that among the shepherd dogs, the prevalence of *Dirofilaria immitis* and age was statistically significant (*P* < 0.05). But among stray dogs, the relationship between age and prevalence of *Dirofilaria* was not significant (*P* > 0.05).

All shepherd dogs were male. Chi square test showed that among stray dogs, *Dirofilaria immitis* infection with dog gender was not statistically significant (*P* > 0.05).

According to the results of this study, out of 99 dogs, 82 and 17 dogs were native and hybrid breeds, respectively. 21 cases of native breed dogs (25.6%) and 9 cases of hybrid dogs (52.9%) were infected. Fisher’s exact test showed that the prevalence of *Dirofilaria immitis* with dog breeds was not significant (*P* = 1), however, among stray dogs, the relationship between breed and the prevalence of parasite infection was statistically significant (*P* = 0.044) (Table 1).

In the present study, none of the stray dogs used anti-parasitic drugs. Fischer’s exact test showed that among the shepherd dogs, the prevalence of *Dirofilaria immitis* with usage anti-parasitic drug was not statistically significant (*P* = 1).

## Discussion

Dirofilariasis is a global disease and in recent decades has been a significant increase in its geographical range in different geographical areas. Therefore, it requires fundamental attention to accurately diagnose and clarify the true epidemiological aspects of it. Among the various diagnostic methods of this disease, the use of microscopic methods and observation of microfilariae in the blood still a reliable method (Hou et al. 2011).

In this study, the prevalence of *Dirofilaria immitis* parasite in two groups of shepherd and stray dogs in Iranshahr city was investigated that prevalence of *D. immitis* was 30.3%, so that the infection rate among the shepherd dogs and stray dogs were 14.3% (7 cases) and 46.0% (23 cases), respectively. Reports about the prevalence of dirofilariasis of dogs in different parts of Iran as follows: in Tehran 1.4% (Meshki and Eslami 2001), Tonekabon 16.25% (RanjbarBahadori et al. 2005), Mashhad 6.6% (Razmi 1999), Garmsar 29.12% (RanjbarBahadori and Hekmatkhah 2007), 12% in Khuzestan province(RanjbarBahadori et al. 2009), 15.38% in Golestan province, 7.69% in Mazandaran province, 51.42% in Gilan province (Malmasi et al. 2011), Kermanshah 18.3% (Bohloli Oskoii et al. 2013) and 11.6% in Tabriz (Hosseinzadeh Varjoy et al. 2016). Due to high prevalence, dirofilariasis is an endemic disease in Iran. The prevalence of dirofilariasis among 886 dogs in China, 16.6% has been reported (Hou et al. 2011). In Brazil, the prevalence of dirofilariasis was reported in 188 dogs using the completed Knott test, 34.45% of dogs were infected (Furtado et al. 2009). The average infection rate of this parasite in American continent was 17.7% (DeFrancesco et al. 2001). Contamination of this nematode has been reported from European countries, especially Spain, Portugal, France and Italy (Ettinger and Feldman 2005). In Asia, the highest levels of infection have been reported in Japan. This nematode is known as most important parasitic infection of dogs in Japan (Perret-Court et al. 2009).

Our results showed that the prevalence of dirofilariasis increased with age, and there was a significant association between dirofilariasis and age among the shepherd dogs. But there was no significant relationship between dirofilariasis and age among stray dogs. In some studies, no significant relationship was found between age and prevalence of *Dirofilariaimmitis* (Nematollahi and Barazandeh 2010; Paykari et al. 2013; Razi Jalali et al. 2010). But in some studies, significant relationship have been reported (Bokai et al. 1998; Meshki 2000; Vieira et al. 2014). The gradual increase of contamination associated with increase age of dogs, may be due to the increase possibility of bites by mosquitoes, prolonged infectious conditions, prolonged proliferation of microfilariae in the blood and insufficient immunity against adult parasites (Bohloli Oskoii et al. 2013; Razmi 1999).

In the present study, all shepherd dogs were male. But in stray dogs group, there was no significant relationship between the prevalence of dirofilariasis and gender (*P* = 0.075). Such findings have been reported in many studies conducted in Iran and other countries (Akhtardanesh et al. 2011; Bokai et al. 1998; Byeon et al. 2007; Furtado et al. 2009; Hou et al. 2011; Malmasi et al. 2011; Nematollahi and Barazandeh 2010; Ranjbar-Bahadori et al. 2011; RanjbarBahadori et al. 2009; RanjbarBahadori and Hekmatkhah 2007; Razi Jalali et al. 2010; Simsek et al. 2008; Tasic et al. 2008; Vieira et al. 2014; Yildirim et al. 2007). However, in some studies (Boonyapakorn et al. 2008; Cringoli et al. 2001; Souza et al. 1997), there was a significant association between the prevalence of *D. immitis* and male gender, so that the prevalence of *D. immitis* among male dogs was higher than female, mainly due to the effect of sex hormones on the contamination and the tendency of dog owners that male dogs are responsible for the protection and safety of home (RanjbarBahadori et al. 2005).

The results of this study indicate that among shepherd dogs, the prevalence of dirofilariasis is not related to dog breeds. However, in stray dogs there was a significant relationship between prevalence of *D. immitis* and breed. The results of the survey that conducted in Ahvaz (Razi Jalali et al. 2010) showed that the prevalence of dirofilariasis in dogs according to native and hybrid breed were 21.4% and 19.6%, respectively. Most dogs in this study were native and hybrid, and no significant relationship was found between breed of dogs and infection rate. Also, there were no significant association between the prevalence of dirofilariasis and breed of dogs in other regions of Iran such as Golestan, Khuzestan and Garmsar and Ahvaz (RanjbarBahadori et al. 2009; RanjbarBahadori and Eslami 2007; RanjbarBahadori and Hekmatkhah 2007).

In this study, none of the stray dogs had received anti-parasitic drugs. Despite the fact that 28 of the 49 shepherd dogs received anti-parasitic drugs, the results showed that there was no significant relationship between the prevalence of dirofilariasis and Usage of anti-parasitic drugs among shepherd dogs. But it is quite obvious that taking anti-parasitic drugs, will reduce infection with microfilariae of *Dirofilaria immitis* (Taylor et al. 2007).

## Conclusion

Given the ever-increasing advances made by humans in all areas, especially public health, due to providing effective control and precautionary measures, but the result of the present study suggest that dirofilariasis disease still exist in Iran, especially is as suspected disease among dogs in Sistan and Balouchestan province. Except for limited studies, no action has been taken in relation to the eradication of this disease, so dirofilariasis disease is a suspected disease among dogs in Sistan and Balouchestan province. Therefore, due to the presence of infection with *Dirofilaria immitis* parasites in shepherd and stray dogs of Iranshahr, veterinarians and public health experts should consider this disease and take necessary measures to control the disease, including the use of anti-parasitic drugs for prevention and treatment.
